# Aging Intensity for Step-Stress Accelerated Life Testing Experiments

**DOI:** 10.3390/e26050417

**Published:** 2024-05-13

**Authors:** Francesco Buono, Maria Kateri

**Affiliations:** Institute of Statistics, RWTH Aachen University, 52062 Aachen, Germany; buono@isw.rwth-aachen.de

**Keywords:** cumulative exposure, cumulative Kulback–Leibler divergence, exponential distribution, kernel density estimation, Kulback–Leibler divergence, maximum likelihood estimation, Weibull distribution, tampered failure rate

## Abstract

The aging intensity (AI), defined as the ratio of the instantaneous hazard rate and a baseline hazard rate, is a useful tool for the describing reliability properties of a random variable corresponding to a lifetime. In this work, the concept of AI is introduced in step-stress accelerated life testing (SSALT) experiments, providing new insights to the model and enabling the further clarification of the differences between the two commonly employed cumulative exposure (CE) and tampered failure rate (TFR) models. New AI-based estimators for the parameters of a SSALT model are proposed and compared to the MLEs in terms of examples and a simulation study.

## 1. Introduction

Conducting tests under normal operating conditions (NOCs) for obtaining information on the lifetimes of highly reliable products is unrealistic, since they require a very long duration, which is usually not possible or economical. For this, accelerated life testing (ALT) experiments are designed and applied to obtain information about the data in a reasonable time frame. With ALT, the test units are exposed to a specific failure-relevant stress (e.g., temperature, pressure, or use rate) and operate in an environment with stronger testing conditions than NOCs. The increased stress reduces the time to failure and produces more observations during the conducted life test. The data collected with an ALT test and the corresponding inferential results have to be eventually extrapolated to NOCs to estimate the lifetime distribution of interest. For some references and the introduction of ALT, see Nelson [1], Meeker and Escobar [2], and Bagdonavicius and Nikulin [3]. The most commonly used, in-practice type of ALT experiment is constant stress ALT (CSALT), under which independent samples of testing units are exposed to different levels of the stress factor and held constant during the whole duration of the experiment (see, for instance, Nelson [4] and Yang [5]). An alternative type of ALT experiment is step-stress ALT (SSALT). A SSALT experiment uses a single sample, and all test units are exposed to exactly the same experimental conditions, which vary (usually in an increasing manner) during the test procedure. In particular, all items are exposed to the same stress level, which is increased once or several times, after a prefixed time or upon the occurrence of a predetermined number of failures. SSALT experiments lead to quicker results than CSALT (see Miller and Nelson [6] and Han and Ng [7]). SSALT tests that involve only a single stress-change point are called simple SSALT.

Statistical inference for SSALT models has been developed for various modeling setups and underlying distributional assumptions, mainly through maximum likelihood or Bayesian procedures. The approaches discussed here are frequentist approaches. For Bayesian treatments of SSALT models, we refer to [8,9,10,11,12,13] and the references cited therein. It is worth noting that the first Bayesian analysis of SSALT models was by DeGroot and Goel [14]. SSALT models are analyzed in terms of the cumulative density function (CDF), the probability density function (PDF), the survival function (SF), and the hazard rate (HR). In reliability context, the concept of aging intensity (AI) has been introduced by Jiang et al. [15] as the ratio of the instantaneous hazard rate and a baseline hazard rate. It is a useful tool to describe reliability properties of a random variable, and it is also used in order to make comparisons among different distributions. However it has never been considered in the SSALT context.

In this work, we introduced an AI function for SSALT and explored its properties and utility. Comparing the cumulative exposure (CE) and tampered failure rate (TFR) models for SSALT through AI, we gained a deeper understanding of their differences in terms of their aging effects on test units. In Section 2, we provide the preliminaries of the SSALT models and set notation. The AI function for SSALT models is introduced and explored for common families of lifetime distributions in Section 3. An AI-based estimation procedure for SSALT models is discussed in Section 4, along with examples, while the AI-based estimation is compared to the maximum likelihood estimation using Kullback–Leibler and cumulative Kullback–Leibler divergence in Section 5. The final section, Section 6, summarizes our findings.

## 2. Preliminaries of SSALT

Consider an SSALT experiment with *m* stress levels, $x_{1},\ldots ,x_{m}$, and *n* units being tested. The stress levels are changed from $x_{j}$ to $x_{j+1}$, j=1,…,m−1, at pre-specified time points $\tau_{1}<\cdots <\tau_{m-1}$. In case of simple SSALT (m=2), the single $\tau_{1}$ is for simplicity and is denoted by τ. Different distributions are assumed in the steps, corresponding to units of behavior under different stress levels, which are, nonetheless, linked in a manner so that the compound CDF is absolutely continuous over all stress levels. Thus, the units’ failure times $T_{1},\ldots ,T_{n}$ are described by the order statistics (OSs) $T_{1:n}\leq \cdots \leq T_{n:n}$ from an absolutely continuous CDF *G*. The model usually employed for ensuring the continuity of *G* is the cumulative exposure (CE) model (see, for instance, Nelson [1,16], Meeker and Escobar [2], Gouno and Balakrishnan [17], Bagdonavicius and Nikulin [3], and Balakrishnan [18]). Furthermore, ALT experiments are frequently censored, with Type-I and Type-II censoring being the most commonly considered censoring schemes. For simplicity of presentation, we restricted the presentation to simple SSALT models and complete observed sample, but our results are extendable to models with m>2 and under censoring.

The CE simple SSALT model is defined by the following CDF:

(1) $$G\left(t\right)=\left\{\begin{array}{cc} F_{1}\left(t\right), & \mathrm{if}\;0<t\leq \tau \\ F_{2}\left(t+c-\tau \right), & \mathrm{if}\;t>\tau \end{array}\right.,$$

where $F_{1}$ and $F_{2}$ are the lifetime CDFs under stress levels $x_{1}$ and $x_{2}$, respectively, and the constant *c* is chosen so that $F_{2}\left(c\right)=F_{1}\left(\tau \right)$, i.e., it ensures the continuity of *G*. Then, the corresponding PDF and HR function are given by

$$g\left(t\right)=\left\{\begin{array}{cc} f_{1}\left(t\right), & \mathrm{if}\;0<t\leq \tau \\ f_{2}\left(t+c-\tau \right), & \mathrm{if}\;t>\tau \end{array}\right.,$$

and

$$h\left(t\right)=\left\{\begin{array}{cc} \frac{f_{1}\left(t\right)}{1-F_{1}\left(t\right)}, & \mathrm{if}\;0<t\leq \tau \\ \frac{f_{2}\left(t+c-\tau \right)}{1-F_{2}\left(t+c-\tau \right)}, & \mathrm{if}\;t>\tau \end{array}\right.,$$

respectively.

A common distributional assumption for the lifetimes under each stress level is the family of exponential distributions, which leads to explicit maximum likelihood estimators (MLEs) for the model parameters. Consider, thus, the CE SSALT model for an exponential distribution, i.e., let $F_{i}∼Exp\left(\theta_{i}\right)$, i=1,2. Then, $c=\frac{\theta_{2}}{\theta_{1}}\tau$, and the CDF of the model is as follows:

(2) $$\begin{array}{ccc} G(t) & = & \left\{\begin{array}{cc} 1-\exp\left(-\frac{t}{\theta_{1}}\right), & \mathrm{if}\;0<t\leq \tau \\ 1-\exp\left(-\frac{t-\left(1-\frac{\theta_{2}}{\theta_{1}}\right)\tau }{\theta_{2}}\right), & \mathrm{if}\;t>\tau \end{array}\right.,\\ & = & \left\{\begin{array}{cc} 1-\exp\left(-\frac{t}{\theta_{1}}\right), & \mathrm{if}\;0<t\leq \tau \\ 1-\exp\left(-\frac{\tau }{\theta_{1}}-\frac{t-\tau }{\theta_{2}}\right), & \mathrm{if}\;t>\tau \end{array}\right.. \end{array}$$

For this model, the maximum likelihood estimators (MLE) for the parameters $\theta_{1}$ and $\theta_{2}$ have been derived and studied, as reviewed and discussed by Balakrishnan [18] and Kateri and Kamps [19], where the unbiasedness of the estimator for $\theta_{2}$ was also proved.

Suppose we have a random sample of lifetimes under (2) of size *n*, with the corresponding OSs $T_{1:n}\leq \cdots \leq T_{n:n}$, such that $n_{1}$ failures are realized in the first stress level, i.e., $T_{n_{1}:n}\leq \tau$, and the remaining $n_{2}$ ($=n-n_{1}$) in the second one. Then, the MLEs for $\theta_{1}$ and $\theta_{2}$ are given by

(3) $$\begin{array}{c} \theta_{1}^{\left(MLE\right)}=\frac{\sum_{i=1}^{n_{1}}T_{i:n}+\left(n-n_{1}\right)\tau }{n_{1}} \end{array}$$

and

(4) $$\begin{array}{c} \theta_{2}^{\left(MLE\right)}=\frac{\sum_{i=n_{1}+1}^{n}T_{i:n}-n_{2}\tau }{n_{2}}, \end{array}$$

respectively.

For a simple SSALT model, Bhattacharyya and Soejoeti [20] were the first to consider a model assumption, alternative to CE, in which the increase in stress level from $x_{1}$ to $x_{2}$ has a multiplicative effect on the subsequent HR, i.e.,

(5) $$h_{2}\left(t\right)=\alpha h_{1}\left(t\right),\;\;t>0,\;\alpha >0\;.$$

This model is known as the tampered failure rate model (TFR), and its CDF is expressed as follows:

(6) $$G\left(t\right)=\left\{\begin{array}{cc} F_{1}\left(t\right), & \mathrm{if}\;0<t\leq \tau \\ 1-{\bar{F}}_{1}^{1-\alpha }\left(\tau \right)\cdot {\bar{F}}_{1}^{\alpha }\left(t\right), & \mathrm{if}\;t>\tau \end{array}\right.,$$

while the corresponding PDF is given by

(7) $$g\left(t\right)=\left\{\begin{array}{cc} f_{1}\left(t\right), & \mathrm{if}\;0<t\leq \tau \\ \alpha {\left[\frac{{\bar{F}}_{1}\left(t\right)}{{\bar{F}}_{1}\left(\tau \right)}\right]}^{\alpha -1}f_{1}\left(t\right), & \mathrm{if}\;t>\tau \end{array}\right..$$

From the expressions in (6) and (7), it is easy to verify that α represents the proportionality constant between the hazard on the first and the second level, i.e., the derivation of (5).

The TFR SSALT model is appealing in a reliability context, being linked through (5) to Cox’s proportional hazard model. Compared to the CE model, it is more convenient to work with when the underlying distributions are something other than exponential (e.g., Weibull), while the TFR and CE models coincide in the case of exponential distributed lifetimes. For a detailed comparison of these two models, we refer to Kateri and Kamps [21].

An equivalent expression for the CDF of the TFR simple SSALT model, which is helpful for deriving statistical inference, is

$$G\left(t\right)=\left\{\begin{array}{cc} F_{1}\left(t\right), & \mathrm{if}\;0<t\leq \tau \\ 1-\frac{1-F_{1}\left(\tau \right)}{1-F_{2}\left(\tau \right)}\left(1-F_{2}\left(t\right)\right), & \mathrm{if}\;t>\tau \end{array}\right..$$

Furthermore, Kateri and Kamps [21] extended the TFR SSALT models to a generalized failure rate-based family of models by considering a flexible-scale family of distributions for the lifetimes under each stress level j∈{1,2}, and it is defined by

(8) $$F_{j}\left(t\right)=1-\exp\left(-\frac{g_{j}\left(t\right)}{\theta_{j}}\right),\;\;t\in \left(0,+\infty \right)$$

where $g_{j}$ is strictly increasing and differentiable on $\left(g_{j}^{-1}\left(0\right),+\infty \right)$, $\theta_{j}>0$ and $\lim_{t\to +\infty }g_{j}\left(t\right)=+\infty$. Standard families of distributions used for modeling lifetimes (e.g., exponential, Weibull, Lomax, Pareto) are members of this family and can be treated in a unified manner (see Table 1 in [21]). This model generalizes TFR in the sense that it can consider different distributional families for the stress levels ($g_{1}\neq g_{2}$), while α in (5) can be non-constant, i.e, time dependent ($\alpha_{t}$).

## 3. AI Function for SSALT

For a non-negative and absolutely continuous random variable *X* with PDF f(·), CDF F(·), survival function (SF) $\bar{F}\left(\cdot \right)$, and hazard rate function h(·), the aging intensity (AI) function is defined as

(9) $$AI\left(t\right)=\frac{h(t)}{\frac{1}{t}\int_{0}^{t}h\left(x\right)dx}=\frac{-th(t)}{\log\bar{F}\left(t\right)}=\frac{-tf(t)}{\bar{F}\left(t\right)\log\bar{F}\left(t\right)}\;,\;\;\;t\in \left(0,+\infty \right)\;,$$

where log denotes the natural logarithm. Thus, AI(t) is the ratio of the instantaneous hazard rate h(t) to the average hazard rate in the interval (0,t) and expresses the units’ average aging behavior. It analyzes the aging property quantitatively, in the sense that the larger the AI, the stronger the tendency of aging. We remark that the survival and the hazard rate functions uniquely determine the AI function, but the converse does not hold. In fact, the AI function of a non-negative random variable determines a family of survival functions through a relation presented in [22]. Some properties of AI functions are presented in Nanda et al. [23], who defined, in particular, a new stochastic order (aging intensity order) based on the AI functions. For further properties, applications, and extensions of the aging intensity functions, see [24,25,26,27,28,29].

So far, the aging intensity has been considered for random variables with an absolutely continuous PDF. In order to extend the notion of the AI function to SSALT models, we need to take into account that the PDF (and consequently the HR function) changes at the stress change point τ. Hence, on the first level, the aging intensity function has the same expression as in (9) but in terms of $h_{1}\left(\cdot \right)$, while on the second level, in order to have the average of the hazard rate in the interval (0,t), we need to consider the change of the hazard at τ. Thus, the aging intensity function for simple SSALT is

$$AI\left(t\right)=\left\{\begin{array}{cc} AI_{1}\left(t\right), & \mathrm{if}\;0<t\leq \tau \\ AI_{2}\left(t\right), & \mathrm{if}\;t>\tau \end{array}\right.,$$

where

(10) $$AI_{1}\left(t\right)=\frac{h_{1}\left(t\right)}{\frac{1}{t}\int_{0}^{t}h_{1}\left(x\right)dx}=\frac{-th_{1}\left(t\right)}{\log{\bar{F}}_{1}\left(t\right)}=\frac{-tf_{1}\left(t\right)}{{\bar{F}}_{1}\left(t\right)\log{\bar{F}}_{1}\left(t\right)}\;,$$

and

(11) $$AI_{2}\left(t\right)=\frac{h_{2}\left(t\right)}{\frac{1}{t}\left(\int_{0}^{\tau }h_{1}\left(x\right)dx+\int_{\tau }^{t}h_{2}\left(x\right)dx\right)}=\frac{-th_{2}\left(t\right)}{\log\left(\frac{{\bar{F}}_{1}\left(\tau \right)}{{\bar{F}}_{2}\left(\tau \right)}\right)+\log{\bar{F}}_{2}\left(t\right)}\;.$$

Consider the CE SSALT model for an exponential distribution with a CDF given in (2). Hence, the aging intensity function is expressed on the two levels of stress as

(12) $$AI^{Exp}\left(t\right)=\left\{\begin{array}{cc} \frac{-t\frac{1}{\theta_{1}}}{-\frac{t}{\theta_{1}}}, & \mathrm{if}\;0<t\leq \tau \\ \frac{-t\frac{1}{\theta_{2}}}{-\left(\frac{\tau }{\theta_{1}}+\frac{t-\tau }{\theta_{2}}\right)}, & \mathrm{if}\;t>\tau \end{array}\right.\;\;=\left\{\begin{array}{cc} 1, & \mathrm{if}\;0<t\leq \tau \\ \frac{1}{1-\left(1-\frac{\theta_{2}}{\theta_{1}}\right)\frac{\tau }{t}}, & \mathrm{if}\;t>\tau \end{array}\right..$$

Analogously, consider next the CE SSALT model for Weibull-distributed lifetimes, i.e., let $F_{i}∼W\left(\theta_{i},\delta \right)$, i=1,2, with a common shape parameter δ under both levels. Then, $F_{j}\left(t\right)=1-\exp\left(-\frac{t^{\delta }}{\theta_{j}}\right)$, j=1,2, leading to $c=\frac{\theta_{2}}{\theta_{1}}\tau$, and the CDF (1) takes the form

$$G\left(t\right)=\left\{\begin{array}{cc} 1-\exp\left(-\frac{t^{\delta }}{\theta_{1}}\right), & \mathrm{if}\;0<t\leq \tau \\ 1-\exp\left(-\frac{1}{\theta_{2}}{\left(t-\left(1-\frac{\theta_{2}}{\theta_{1}}\right)\tau \right)}^{\delta }\right), & \mathrm{if}\;t>\tau \end{array}\right.,$$

from which the PDF and the hazard rate function are derived as follows:

$$g\left(t\right)=\left\{\begin{array}{cc} \frac{\delta t^{\delta -1}}{\theta_{1}}\exp\left(-\frac{t^{\delta }}{\theta_{1}}\right), & \mathrm{if}\;0<t\leq \tau \\ \frac{\delta }{\theta_{2}}{\left(t-\left(1-\frac{\theta_{2}}{\theta_{1}}\right)\tau \right)}^{\delta -1}\exp\left(-\frac{1}{\theta_{2}}{\left(t-\left(1-\frac{\theta_{2}}{\theta_{1}}\right)\tau \right)}^{\delta }\right), & \mathrm{if}\;t>\tau \end{array}\right.,$$

and

$$h\left(t\right)=\left\{\begin{array}{cc} \frac{\delta t^{\delta -1}}{\theta_{1}}, & \mathrm{if}\;0<t\leq \tau \\ \frac{\delta }{\theta_{2}}{\left(t-\left(1-\frac{\theta_{2}}{\theta_{1}}\right)\tau \right)}^{\delta -1}, & \mathrm{if}\;t>\tau \end{array}\right.,$$

respectively. Finally, through (10) and (11), the AI function on the two stress levels can be expressed as

(13) $$AI^{CE_{\delta }}\left(t\right)=\left\{\begin{array}{cc} \delta , & \mathrm{if}\;0<t\leq \tau \\ \frac{t\delta }{\left(t-\left(1-\frac{\theta_{2}}{\theta_{1}}\right)\tau \right)}, & \mathrm{if}\;t>\tau \end{array}\right..$$

For the TFR simple SSALT with the scale family of distributions as given in (8), the AI function terms (10) and (11) are simplified to (10), which simplifies to

$$AI_{1}^{TFR}\left(t\right)=-\frac{th_{1}\left(t\right)}{\log{\bar{F}}_{1}\left(t\right)}=\frac{-t\cdot \frac{g_{1}^{\prime }\left(t\right)}{\theta_{1}}}{-\frac{g_{1}\left(t\right)}{\theta_{1}}}=t\cdot \frac{g_{1}^{\prime }\left(t\right)}{g_{1}\left(t\right)}$$

and

$$\begin{array}{ccc} AI_{2}^{TFR}\left(t\right) & = & \frac{-th_{2}\left(t\right)}{\log\left(\frac{{\bar{F}}_{1}\left(\tau \right)}{{\bar{F}}_{2}\left(\tau \right)}\right)+\log{\bar{F}}_{2}\left(t\right)}=\frac{-t\frac{g_{2}^{\prime }\left(t\right)}{\theta_{2}}}{-\frac{g_{1}\left(\tau \right)}{\theta_{1}}-\frac{g_{2}\left(t\right)-g_{2}\left(\tau \right)}{\theta_{2}}}\\ & = & t\cdot \frac{g_{2}^{\prime }\left(t\right)}{\frac{\theta_{2}}{\theta_{1}}g_{1}\left(\tau \right)+g_{2}\left(t\right)-g_{2}\left(\tau \right)}. \end{array}$$

In particular, at the stress change point τ, we have

$$AI_{2}^{TFR}\left(\tau \right)=\frac{\theta_{1}}{\theta_{2}}\tau \frac{g_{2}^{\prime }\left(\tau \right)}{g_{1}\left(\tau \right)}.$$

Hence, if we consider the case in which $g_{1}=g_{2}$, the condition $AI_{2}^{TFR}\left(\tau \right)>AI_{1}^{TFR}\left(\tau \right)$ is equivalent to

$$\frac{\theta_{1}}{\theta_{2}}\tau \frac{g_{1}^{\prime }\left(\tau \right)}{g_{1}\left(\tau \right)}>\tau \frac{g_{1}^{\prime }\left(\tau \right)}{g_{1}\left(\tau \right)},$$

which holds if, and only if, $\theta_{1}>\theta_{2}$. This is a reasonable assumption representing a higher stress in the second level with respect to the first one. Furthermore, when $g_{1}=g_{2}$ and $\theta_{1}=\theta_{2}$ (i.e., constant stress), $AI_{1}^{TFR}=AI_{2}^{TFR}$, as expected.

The $AI_{1}^{TFR}$ and $AI_{2}^{TFR}$ expressions for some distributions of interest, members of the scale family of distributions (8), are as follows:

(i) $Exp(\theta_{1})$, $Exp(\theta_{2})$: $g_{1}\left(t\right)=g_{2}\left(t\right)=t$
  $$AI_{1}^{Exp}\left(t\right)=1\;\mathrm{and}\;AI_{2}^{Exp}\left(t\right)=\frac{t}{t-\left(1-\frac{\theta_{2}}{\theta_{1}}\right)\tau }$$
(ii) $W(\theta_{1},\delta )$, $W(\theta_{2},\delta )$: $g_{1}\left(t\right)=g_{2}\left(t\right)=t^{\delta }$
  (14) $$AI_{1}^{TFR_{\delta }}\left(t\right)=\delta \;\mathrm{and}\;AI_{2}^{TFR_{\delta }}\left(t\right)=\frac{\delta t^{\delta }}{t^{\delta }-\left(1-\frac{\theta_{2}}{\theta_{1}}\right)\tau^{\delta }}$$
(iii) $Gompertz(\theta_{1},d)$, $Gompertz(\theta_{2},d)$: $g_{1}\left(t\right)=g_{2}\left(t\right)=e^{dt}-1$
  $$AI_{1}^{Gomp}\left(t\right)=t\cdot \frac{de^{dt}}{e^{dt}-1}\;\mathrm{and}\;AI_{2}^{Gomp}\left(t\right)=\frac{dt}{1+\frac{\theta_{2}}{\theta_{1}}\left(e^{d(\tau -t)}-e^{-dt}\right)-e^{d(\tau -t)}}$$

**Remark** **1.** *For exponential lifetimes, $AI_{1}^{Exp}$ and $AI_{2}^{Exp}$ (obtained also from (14) for δ=1), obviously coincide with those under the CE model, i.e., (12), as expected, since the TFR and CE are equivalent for exponential distributed lifetimes. For the Weibull distribution, $AI_{2}^{TFR_{\delta }}\left(t\right)$ in (14) can be equivalently written as*

$$AI_{2}^{TFR_{\delta }}\left(t\right)=\frac{\delta }{1-\left(1-\frac{\theta_{2}}{\theta_{1}}\right){\left(\frac{\tau }{t}\right)}^{\delta }}.$$

**Figure 1:**
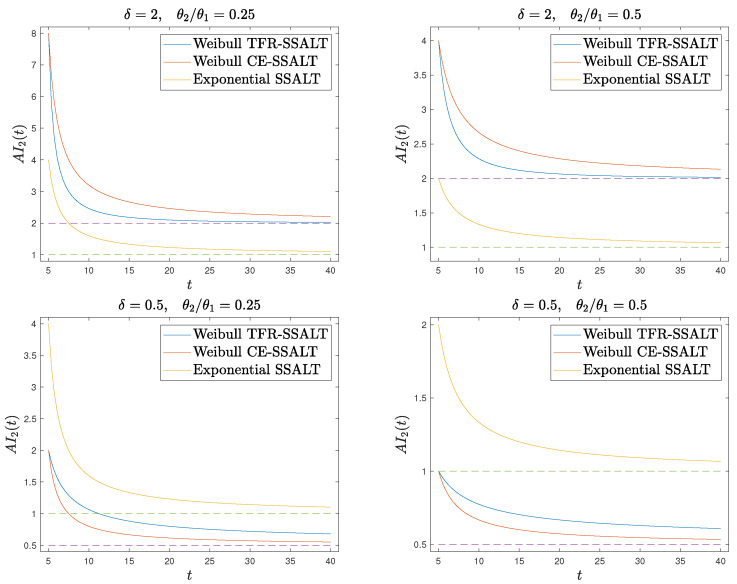
Aging intensity function on the second stress level for the Weibull and exponential distributions for the TFR and CE SSALT models, for δ=2 (**upper**), δ=0.5 (**lower**), $\theta_{2}/\theta_{1}=0.25$ (**left**), and $\theta_{2}/\theta_{1}=0.5$ (**right**). The dashed lines represent the AI of a CSALT model at level $x_{1}$ (truncated at τ, it is also the AI on the first level of a SSALT model, i.e., the constant 1 for the exponential distribution and δ for the Weibull distribution.

In Figure 1, we plot the AI function in the second stress level for the Weibull and the exponential distributions with the TFR and CE SSALT model, for different choices of the parameter δ of the Weibull distribution and of the ratio $\theta_{2}/\theta_{1}$. Recall that in the case of exponential distributed lifetimes, the TFR and CE models coincide. Moreover, we chose values of $\theta_{2}/\theta_{1}$ less than one in order to represent the higher stress in the second level.

For Weibull lifetimes, $AI_{2}^{TFR_{\delta }}$ in (14) and $AI_{2}^{CE_{\delta }}$ in (13) have the same functional form, but the TFR model is in terms of $t^{\delta }$, $\tau^{\delta }$, while the CE is in terms of *t*, τ, respectively. This has as a consequence that for δ>1 (aging effect of exposure to stress), $AI_{2}^{TFR_{\delta }}<AI_{2}^{CE_{\delta }}$, while for δ<1 (recovering effect), $AI_{2}^{TFR_{\delta }}>AI_{2}^{CE_{\delta }}$ (see also Figure 1). Furthermore, at the time point of stress level change τ, we have $AI_{2}^{TFR_{\delta }}\left(\tau \right)=AI_{2}^{CE_{\delta }}\left(\tau \right)=\delta \frac{\theta_{1}}{\theta_{2}}$. Thus, though the instant stress change effect on the AI is the same for both models, the AI development in time differs. Focusing on the aging case, we realize another qualitative difference between the CE and TFR models. The AI curve under a Weibull TFR model is steeper than that of the corresponding CE model, reaching, after some time, the AI value δ of Weibull distributions, which is not the case for the AI under the CE model. For the CE model, AI stabilizes as well but at a higher level. This difference of the two models can motivate the decision for one or the other when modeling SSALT experimental data, depending on the effect that the stress factor has on the aging at a particular application setup.

The AI of a SSALT model can be estimated parametrically. For example, for Weibull lifetimes, we have $AI\left(t\right)=AI\left(t|\theta_{1},\theta_{2},\delta \right)$ and $\hat{AI}\left(t\right)=AI\left(t|{\hat{\theta }}_{1},{\hat{\theta }}_{2},\hat{\delta }\right)$, where ${\hat{\theta }}_{1},{\hat{\theta }}_{2},$ and $\hat{\delta }$ are the maximum likelihood estimators (MLEs) of the parameters.

**Remark** **2.** *We introduced the AI function and explored it for characteristic families of lifetime distributions under CE and TFR assumptions for simple SSALT models. The discussion can be extended to SSALT models for m>2 stress levels. In this case, AI is defined by*

$$\begin{array}{ccc} AI(t) & = & \left\{\begin{array}{cc} \frac{h_{1}\left(t\right)}{\frac{1}{t}\int_{0}^{t}h_{1}\left(x\right)dx}, & \mathrm{if}\;0<t\leq \tau_{1}\\ \frac{h_{i}\left(t\right)}{\frac{1}{t}\left(\sum_{j=1}^{i-1}\int_{\tau_{j-1}}^{\tau_{j}}h_{j}\left(x\right)dx+\int_{\tau_{i-1}}^{t}h_{i}\left(x\right)dx\right)}, & \mathrm{if}\;\tau_{i-1}<t\leq \tau_{i},\;\;2\leq i\leq m \end{array}\right.\\ & = & \left\{\begin{array}{cc} \frac{-tf_{1}\left(t\right)}{{\bar{F}}_{1}\left(t\right)\log{\bar{F}}_{1}\left(t\right)}, & \mathrm{if}\;0<t\leq \tau_{1}\\ \frac{-th_{i}\left(t\right)}{\sum_{j=1}^{i-1}\log\left(\frac{{\bar{F}}_{j}\left(\tau_{j}\right)}{{\bar{F}}_{j+1}\left(\tau_{j}\right)}\right)+\log{\bar{F}}_{i}\left(t\right)}, & \mathrm{if}\;\tau_{i-1}<t\leq \tau_{i},\;\;2\leq i\leq m \end{array}\right. \end{array}$$

*where $\tau_{1}<\cdots <\tau_{m-1}$ are the stress change points, and $\tau_{0}=0$ and $\tau_{m}=+\infty$ by convention. For example, in the case of exponential lifetimes, the CDF of a SSALT model with m stress levels takes the form*

$$G\left(t\right)=\left\{\begin{array}{cc} 1-\exp\left(-\frac{t}{\theta_{1}}\right), & \mathrm{if}\;0<t\leq \tau_{1}\\ 1-\exp\left(-\frac{1}{\theta_{i}}\left(t+\theta_{i}\sum_{j=1}^{i-1}\frac{\tau_{j}-\tau_{j-1}}{\theta_{j}}-\tau_{i-1}\right)\right), & \mathrm{if}\;\tau_{i-1}<t\leq \tau_{i},\;\;2\leq i\leq m \end{array}\right.,$$

*where $\theta_{i}$ is the exponential parameter under level i, for i∈{1,…,m}. The corresponding AI is given by*

(15) $$AI^{Exp}\left(t\right)=\left\{\begin{array}{cc} 1, & \mathrm{if}\;0<t\leq \tau_{1}\\ \frac{t}{t+\theta_{i}\sum_{j=1}^{i-1}\frac{\tau_{j}-\tau_{j-1}}{\theta_{j}}-\tau_{i-1}}, & \mathrm{if}\;\tau_{i-1}<t\leq \tau_{i},\;\;2\leq i\leq m \end{array}\right..$$

*Notice that if m=2, then (15) reduces to (12).*

## 4. AI-Based Estimation

In this section, we propose a method based on the AI function to estimate the parameters $\theta_{1}$ and $\theta_{2}$ in a CE SSALT model for an exponential distribution and compare the results with the those of MLEs.

Let $T_{1:n},\ldots ,T_{n:n}$ be the OSs of a sample from a simple SSALT experiment, such that $n_{1}$ failures are realized at the first stress level, and the remaining $n_{2}$ ($=n-n_{1}$), at the second one (i.e., $T_{n_{1}:n}\leq \tau <T_{n_{1}+1:n}$). The estimation of the corresponding AI function builds on a kernel-based estimation of the model’s CDF *G* and PDF *g*. We proceed by dividing the support in the intervals (0,τ] and (τ,+∞). On the first stress level, i.e., for t∈(0,τ], the CDF is estimated with a kernel approach and the assumption of positive support on the data $T_{1:n},\ldots ,T_{n_{1}:n}$. The obtained CDF is then scaled by the factor $n_{1}/n$, the proportion of failures in the first stress level, which approximates G(τ). The CDF in the second stress level is estimated using the same approach applied on the shifted data $T_{n_{1}+1:n}-\tau ,\ldots ,T_{n:n}-\tau$, which also have a positive support. The derived CDF is scaled by the factor $n_{2}/n$, representing the proportion of failures on the second stress level, and the term $n_{1}/n$ is also added to the estimation of the CDF in order to ensure that it is a CDF, i.e, it fulfills its continuity at τ and is monotone increasing. Along the same lines, the PDF is estimated by applying a kernel density estimation procedure separately to the data observed under the first stress level and the shifted data of the second, synthesizing the derived PDFs in a mixture weighted by the factors $n_{1}/n$ and $n_{2}/n$, respectively.

Denoting the resulting estimators for the PDF and the CDF by $\tilde{g}$ and $\tilde{G}$, respectively, the estimator for the AI function is

(16) $$\tilde{AI}\left(t\right)=\frac{-t\tilde{g}\left(t\right)}{\left(1-\tilde{G}\left(t\right)\right)\log\tilde{G}\left(t\right)}.$$

We remark that we do not need a piecewise function to estimate AI since we work directly with *G* and *g* and not with $F_{1},\;F_{2},\;f_{1}$, and $f_{2}$.

**Figure 2:**
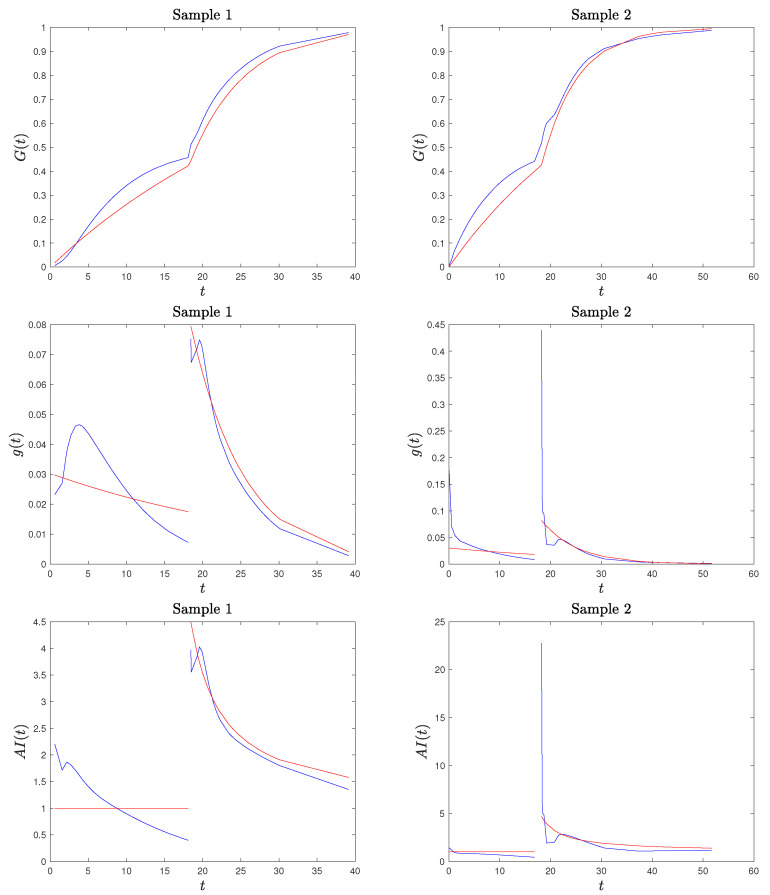
True and estimated CDF, PDF, and AI function in red and blue, respectively, for two simulated samples of the considered SSALT exponential model.

**Example** **1.** *We applied the procedure described above to two randomly generated sample from model (2), with stress change point τ=18.2 and parameters $\theta_{1}=33$ and $\theta_{2}=7$, both of sample size n=80. We present the results in Figure 2 where we compare the kernel-based estimated CDF and PDF and the AI function with the true ones. We consider two generated samples to highlight that the proposed method provides a good estimation of the true functions, but, especially for the PDF and the AI function (see Sample 2), it may have a considerable local error right after the stress change point. Based on this observation, in our procedure described below, we excluded the estimate of the AI function at $T_{n_{1}+1:n}$, as it may cause misleading results (confirmed by our simulation studies).*

**Figure 3:**
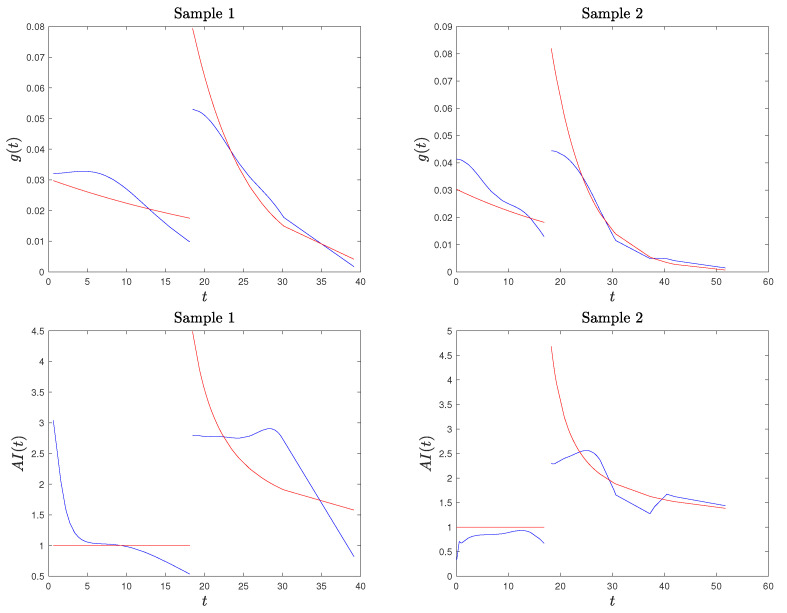
True and estimated PDF and AI function in red and blue, respectively, based on the kernel method with data reflection, for the two simulated samples of Example 1.

**Remark** **3.** Based on our simulation studies, and also supported by Figure 2, the kernel density estimation of the PDF is not fully satisfactory, confirming the well-known problem of kernel density estimation with bounded supports (especially on the lower bound of the second stress level). A method to deal with the problem of bounded support S is the reflection technique for kernel density estimation (see Silverman [30], chapter 2.10). The method consists of augmenting the data by adding the reflections of all points in the boundary, deriving the PDF estimation on an unbounded support and based on 2n data points, and then obtaining the estimated PDF for the original data on S as twice the above-derived PDF. We adapted this method to a SSALT model by dividing the sample data from the first and second level. For the first stress level and its lower bound of 0, the reflections of $T_{1},\ldots ,T_{n_{1}}$ are $-T_{1},\ldots ,-T_{n_{1}}$, the kernel density estimator $f^{*}$ is constructed using 2n points, and the estimated PDF for the original data is given by ${\tilde{f}}_{1}\left(t\right)=2f_{1}^{*}\left(t\right)I\left(t>0\right)$, where I(·) is the indicator function. For the second level, we reflect the data with respect to τ, consider, additionally, the points $2\tau -T_{i:n}$, $i\in \{n_{1}+1,\ldots ,n\}$, derive the kernel density estimated $f_{2}^{*}$, and set ${\tilde{f}}_{2}\left(t\right)=2f_{2}^{*}\left(t\right)I\left(t>\tau \right)$. Then, the PDF of the SSALT model is estimated by $\tilde{f}\left(t\right)=\frac{n_{1}}{n}{\tilde{f}}_{1}\left(t\right)+\frac{n_{2}}{n}{\tilde{f}}_{2}\left(t\right)$. The results obtained in this way seem to be much better with regard to the PDF, but they are not satisfying when considering the corresponding estimated AI function (which depends also on the estimated CDF). In Figure 3, we present the plots for the estimated PDF and AI function for the same two samples of Example 1. Hence, we decided to proceed with the PDF estimated using the classical kernel approach and excluding the AI estimate at point $T_{n_{1}+1:n}$.

We now introduce new estimators for the parameters $\theta_{1}$ and $\theta_{2}$, alternative to the MLEs, that are based on the kernel-based estimated AI function. They are derived by minimizing the distance between the estimated AI (16), evaluated at the order statistics of the sample, $T_{1:n},\ldots ,T_{n:n}$, by excluding $T_{n_{1}+1:n}$, and the theoretical values of the aging intensity at these points given in (12) by assuming a SSALT model for exponential distributed lifetimes with parameters $\theta_{1}$ and $\theta_{2}$. More precisely, the function to be minimized is

(17) $$H^{*}\left(\theta_{1},\theta_{2}\right)=\sum_{i=1}^{n_{1}}{\left(\tilde{AI}\left(T_{i:n}\right)-1\right)}^{2}+\sum_{i=n_{1}+2}^{n}{\left(\tilde{AI}\left(T_{i:n}\right)-\frac{1}{1-\left(1-\frac{\theta_{2}}{\theta_{1}}\right)\frac{\tau }{T_{i:n}}}\right)}^{2}.$$

Hence, we need to solve the system

(18) $$\frac{\partial H^{*}\left(\theta_{1},\theta_{2}\right)}{\partial \theta_{1}}=0\;\mathrm{and}\;\frac{\partial H^{*}\left(\theta_{1},\theta_{2}\right)}{\partial \theta_{2}}=0\;,$$

where

$$\begin{array}{ccc} \frac{\partial H^{*}\left(\theta_{1},\theta_{2}\right)}{\partial \theta_{1}} & = & 2\sum_{i=n_{1}+2}^{n}\left(\tilde{AI}\left(T_{i:n}\right)-\frac{1}{1-\left(1-\frac{\theta_{2}}{\theta_{1}}\right)\frac{\tau }{T_{i:n}}}\right)\cdot \frac{\frac{-\theta_{2}\tau }{\theta_{1}^{2}T_{i:n}}}{{\left(1-\left(1-\frac{\theta_{2}}{\theta_{1}}\right)\frac{\tau }{T_{i:n}}\right)}^{2}},\\ \frac{\partial H^{*}\left(\theta_{1},\theta_{2}\right)}{\partial \theta_{2}} & = & 2\sum_{i=n_{1}+2}^{n}\left(\tilde{AI}\left(T_{i:n}\right)-\frac{1}{1-\left(1-\frac{\theta_{2}}{\theta_{1}}\right)\frac{\tau }{T_{i:n}}}\right)\cdot \frac{\frac{\tau }{\theta_{1}T_{i:n}}}{{\left(1-\left(1-\frac{\theta_{2}}{\theta_{1}}\right)\frac{\tau }{T_{i:n}}\right)}^{2}}. \end{array}$$

Note that the first sum in (17) is constant with respect to $\theta_{1}$ and $\theta_{2}$, so it may seem that $T_{1:n},\ldots ,T_{n_{1}:n}$ (and also $T_{n_{1}+1:n}$, excluded from the second sum) are not involved in the derivation of the estimators, but they actually influence the values of the aging intensity estimates in $T_{n_{1}+1:n},\ldots ,T_{n:n}$ through the estimated PDF and CDF. Moreover, the system in (18) is not analytically solvable, and numerical methods have to be applied. Hence, we minimize (17) numerically by choosing as initial values for $\theta_{1}$ and $\theta_{2}$ the maximum likelihood estimates given in (3) and (4). The estimators obtained in this way are denoted by ${\overset{ˇ}{\theta }}_{1}$ and ${\overset{ˇ}{\theta }}_{2}$.

**Example** **2.** *Consider the setup described in Example 1 with n=80, τ=18.2, $\theta_{1}=33$, and $\theta_{2}=7$. We performed a simulation study and derive the estimates for $\theta_{1}$ and $\theta_{2}$ based on the AI function and the MLEs for 10.000 simulated samples. We observed that the ${\overset{ˇ}{\theta }}_{1}$’s estimates of $\theta_{1}$ based on the AI are closer than the corresponding MLEs’ ${\hat{\theta }}_{1}$ to the real value of $\theta_{1}$ in 60.2% of the cases, while this holds for $\theta_{2}$ in 43.0% of the simulated samples. In order to perform a comparison based on both $\theta_{1}$ and $\theta_{2}$, we checked that the following inequality based on relative errors is satisfied in 59.0% of the samples:*

$$\frac{|{\overset{ˇ}{\theta }}_{1}-\theta_{1}|}{\theta_{1}}+\frac{|{\overset{ˇ}{\theta }}_{2}-\theta_{2}|}{\theta_{2}}<\frac{|{\hat{\theta }}_{1}-\theta_{1}|}{\theta_{1}}+\frac{|{\hat{\theta }}_{2}-\theta_{2}|}{\theta_{2}}$$

**Figure 4:**
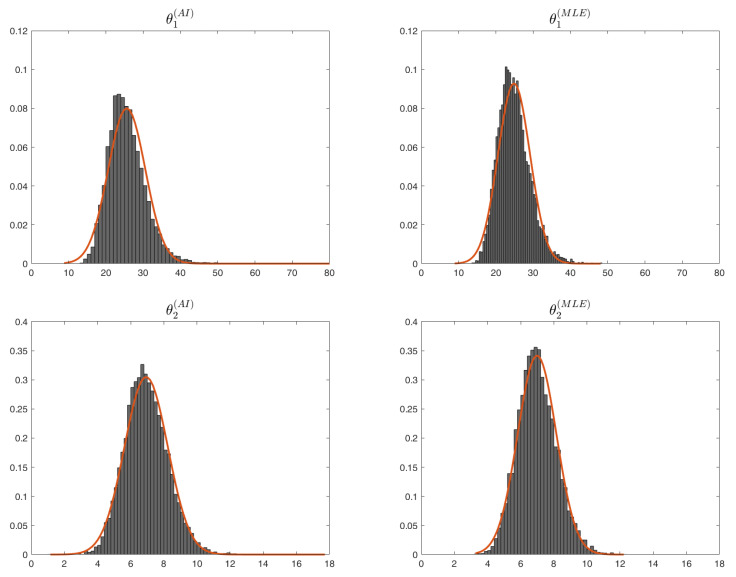
Histograms with the estimates of $\theta_{1}$ and $\theta_{2}$ based on AI and MLE (${\overset{ˇ}{\theta }}_{i}=\theta_{i}^{\left(AI\right)}$, ${\hat{\theta }}_{i}=\theta_{i}^{\left(MLE\right)}$, i=1,2).

*The mean observed bias for the estimates of $\theta_{1}$ based on the AI function and the maximum likelihood are given by 7.3992 and 8.1316, respectively, while for $\theta_{2}$, we have 0.0698 (AI) and 0.0151 (MLE). Recall that the ${\hat{\theta }}_{2}$ is unbiased. In Figure 4, we plot the histograms with the estimates for $\theta_{1}$ and $\theta_{2}$, both based on AI and MLE, showing also the PDF of the corresponding fitted normal distributions. The estimated means and standard deviations of the fitted normal distributions are given in Table 1.*

Next, we consider an example of a SSALT model with m=3, and we derive new estimators for the parameters $\theta_{1}$, $\theta_{2}$, and $\theta_{3}$ based on the AI function and on minimizing a function analogous to $H^{*}$ in (17) for the case of m=2.

**Figure 5:**
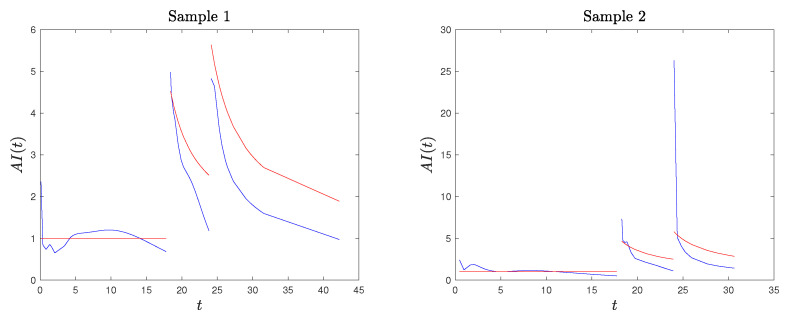
True and estimated AI function in red and blue, respectively, for two simulated samples of the considered SSALT exponential model with three stress levels.

**Example** **3.** *Consider a CE SSALT model for exponential lifetimes under each stress level, with stress change points $\tau_{1}=18.2$ and $\tau_{2}=24$, parameters $\theta_{1}=33$, $\theta_{2}=7$, and $\theta_{3}=3$, and with sample size n=80. First, we compare the kernel-based estimated AI function with the true one given in (15) in the case of m=3. We show the results for two generated samples to remark that the proposed method provides a good estimation of the true function, but it may have a considerable local error right after the second stress change point (see Sample 2). The results are shown in Figure 5. Based on this observation, which is analogous to the observation made in Example 1 for the corresponding simple SSALT, in our procedure, described below, we excluded the estimate of the AI function at one point ($T_{n_{1}+n_{2}+1:n}$) as it may cause misleading results ($n_{1}$ and $n_{2}$ represent the number of failures in the first and second stress levels, respectively).*

*Next, we performed a simulation study and derived the estimates for $\theta_{1}$, $\theta_{2}$, and $\theta_{3}$ based on the AI function and the MLEs for 10.000 simulated samples. In 1% of the cases (103 over 10.000), the optimization procedure did not converge. For those cases, we applied a constrained optimization procedure to our function. We observed that the ${\overset{ˇ}{\theta }}_{1}$’s estimates of $\theta_{1}$ based on the AI are closer than the corresponding MLEs’ ${\hat{\theta }}_{1}$ to the real value of $\theta_{1}$ in 68.1% of the cases, and this holds for $\theta_{2}$ and $\theta_{3}$ in 38.7% and 39.1% of the simulated samples, respectively. In order to perform a comparison based on the three parameters, $\theta_{1}$, $\theta_{2}$, and $\theta_{3}$, we checked that the following inequality based on relative errors was satisfied in 45.7% of the samples:*

$$\frac{|{\overset{ˇ}{\theta }}_{1}-\theta_{1}|}{\theta_{1}}+\frac{|{\overset{ˇ}{\theta }}_{2}-\theta_{2}|}{\theta_{2}}+\frac{|{\overset{ˇ}{\theta }}_{3}-\theta_{3}|}{\theta_{3}}<\frac{|{\hat{\theta }}_{1}-\theta_{1}|}{\theta_{1}}+\frac{|{\hat{\theta }}_{2}-\theta_{2}|}{\theta_{2}}+\frac{|{\hat{\theta }}_{3}-\theta_{3}|}{\theta_{3}}$$

*The estimated means and standard deviations of the fitted normal distributions are given in Table 2. Notice that compared to ${\hat{\theta }}_{1}$, ${\overset{ˇ}{\theta }}_{1}$ has a higher standard deviation but is of reduced bias. For the other two stress levels, the maximmum likelihood estimation seems to behave slightly better for this example (in terms of bias and standard deviation) than the AI based estimation.*

**Figure 6:**
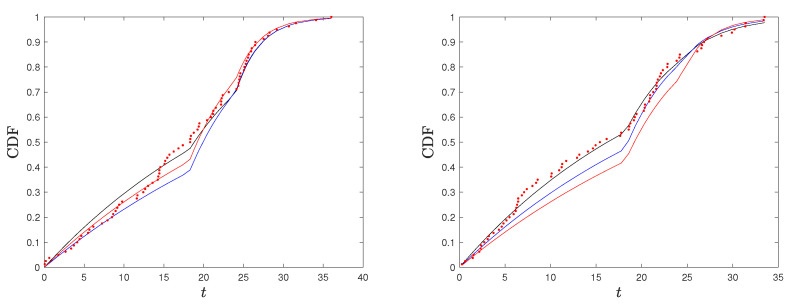
True CDF (red), estimated CDFs based on MLE (black), and AI (blue) with points corresponding to empirical CDF (red dots) for two simulated samples from Example 3.

*Finally, to compare the two different estimation approaches to the true distribution, in Figure 6, we plot the true CDF jointly with the maximum likelihood and AI-based estimated CDFs, along with the points corresponding to the empirical CDF for two of the simulated samples. In Figure 6 (left), we have a case in which the estimated CDF based on the AI and the one based on the MLE are both close to the underlying true distribution. They are indistinguishable on the third level, while the one based on AI (MLE) performs slightly better on the first (second) level. In Figure 6 (right), we have a case in which the simulated data are far from the true distribution, and the estimated CDF based on the AI is closer to the true CDF compared with the one based on the MLE, seeming to be more robust to outliers.*

## 5. Goodness-of-Fit Testing for SSALT

In this section, we cover the goodness-of-fit testing for the SSALT model with exponential distributed lifetimes based on the Kullback–Leibler (KL) divergence. The Kullback–Leibler divergence between two probability density functions *f* and *g* with non-negative support is defined as follows (see Kullback and Leibler [31]):

$$KL\left(f,g\right)=\int_{0}^{+\infty }f\left(x\right)\log\frac{f(x)}{g(x)}dx,$$

It is non-negative and equal to 0 if, and only if, f=g almost everywhere. The KL divergence is not symmetric in *f* and *g* so that KL(f,g)≠KL(g,f).

The PDF of the exponential SSALT model is

$$g\left(t\right)=g\left(t|\theta_{1},\theta_{2}\right)=\left\{\begin{array}{cc} \frac{1}{\theta_{1}}\exp\left(-\frac{t}{\theta_{1}}\right), & \mathrm{if}\;0<t\leq \tau ,\\ \frac{1}{\theta_{2}}\exp\left(-\frac{1}{\theta_{2}}\left(t+\frac{\theta_{2}}{\theta_{1}}\tau -\tau \right)\right), & \mathrm{if}\;t>\tau . \end{array}\right.$$

The AI-based estimated PDF is then $\overset{ˇ}{g}\left(t\right)=g\left(t|{\overset{ˇ}{\theta }}_{1},{\overset{ˇ}{\theta }}_{2}\right)$, t>0, while the maximum likelihood estimated PDF is denoted by $\hat{g}\left(t\right)=g\left(t|{\hat{\theta }}_{1},{\hat{\theta }}_{2}\right)$, t>0. Hence, the KL divergence between the exact theoretical PDF *g* and the AI-based estimated $\overset{ˇ}{g}$ is expressed as follows:

$$\begin{array}{ccc} KL(g,\overset{ˇ}{g}) & = & \int_{0}^{\tau }\frac{1}{\theta_{1}}\exp\left(-\frac{x}{\theta_{1}}\right)\log\left(\frac{{\overset{ˇ}{\theta }}_{1}}{\theta_{1}}\exp\left(-\frac{x}{\theta_{1}}+\frac{x}{{\overset{ˇ}{\theta }}_{1}}\right)\right)dx\\ & & +\int_{\tau }^{+\infty }\frac{1}{\theta_{2}}\exp\left(-\frac{1}{\theta_{2}}\left(x+\frac{\theta_{2}}{\theta_{1}}\tau -\tau \right)\right)\log\left(\frac{{\overset{ˇ}{\theta }}_{2}}{\theta_{2}}\exp\left(-\frac{1}{\theta_{2}}\left(x+\frac{\theta_{2}}{\theta_{1}}\tau -\tau \right)\right.\right.\\ & & \;\;\;\;\;\;\;\;\;\;\left.\left.+\frac{1}{{\overset{ˇ}{\theta }}_{2}}\left(x+\frac{{\overset{ˇ}{\theta }}_{2}}{{\overset{ˇ}{\theta }}_{1}}\tau -\tau \right)\right)\right)dx. \end{array}$$

By integrating by parts in the above integrals, we further obtain

$$\begin{array}{ccc} KL(g,\overset{ˇ}{g}) & = & \left(1-\exp\left(-\frac{\tau }{\theta_{1}}\right)\right)\log\frac{{\overset{ˇ}{\theta }}_{1}}{\theta_{1}}+\theta_{1}\left(\frac{1}{{\overset{ˇ}{\theta }}_{1}}-\frac{1}{\theta_{1}}\right)\left(-\frac{\tau }{\theta_{1}}\exp\left(-\frac{\tau }{\theta_{1}}\right)+1-\exp\left(-\frac{\tau }{\theta_{1}}\right)\right)\\ & & +\left(\log\frac{{\overset{ˇ}{\theta }}_{2}}{\theta_{2}}+\left(\frac{1}{{\overset{ˇ}{\theta }}_{1}}-\frac{1}{\theta_{1}}-\frac{1}{{\overset{ˇ}{\theta }}_{2}}+\frac{1}{\theta_{2}}\right)\tau \right)\exp\left(-\frac{\tau }{\theta_{1}}\right)\\ & & +\theta_{2}\left(\frac{1}{{\overset{ˇ}{\theta }}_{2}}-\frac{1}{\theta_{2}}\right)\exp\left(\left(\frac{1}{\theta_{2}}-\frac{1}{\theta_{1}}\right)\tau \right)\left(\frac{\tau }{\theta_{2}}\exp\left(-\frac{\tau }{\theta_{2}}\right)+\exp\left(-\frac{\tau }{\theta_{2}}\right)\right), \end{array}$$

which is non-negative and equal to 0 if, and only if, $\theta_{1}={\overset{ˇ}{\theta }}_{1}$ and $\theta_{2}={\overset{ˇ}{\theta }}_{2}$. The KL divergence between *g* and the MLE $\hat{g}$ is defined analogously. We remark that the knowledge of *g* and, hence, the evaluations of $KL(g,\overset{ˇ}{g})$ and $KL(g,\hat{g})$ are obtained only with simulated data.

**Figure 7:**
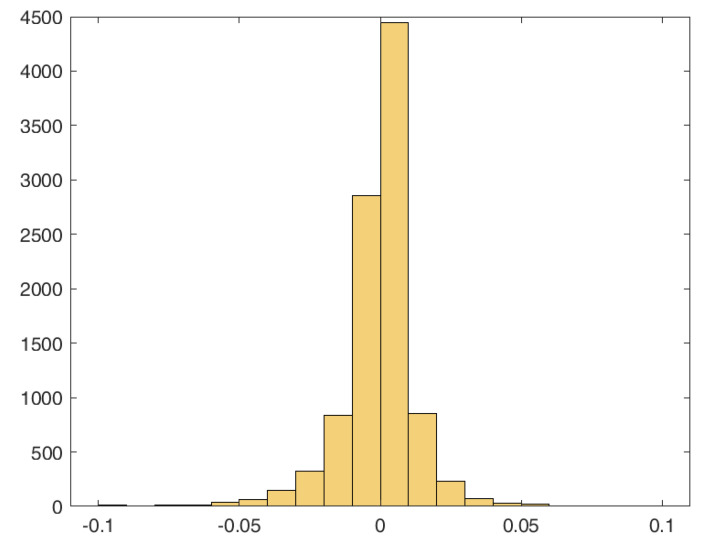
Histogram of the differences $KL\left(g,\hat{g}\right)-KL\left(g,\overset{ˇ}{g}\right)$ based on the simulation study described in Example 2.

**Figure 8:**
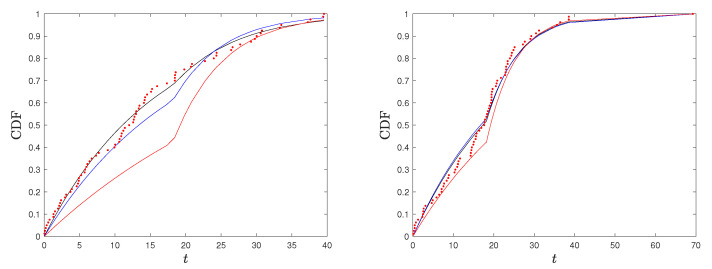
True CDF (red), estimated CDFs based on MLE (black), and AI (blue) with points corresponding to empirical CDF (red dots) for sample with outliers (**left**) and random selected sample (**right**) from Example 2.

Considering the simulation study described in Example 2, we have that the inequality

$$KL\left(g,\overset{ˇ}{g}\right)<KL\left(g,\hat{g}\right)$$

is satisfied in 56.60% of cases, but the differences between $KL(g,\overset{ˇ}{g})$ and $KL(g,\hat{g})$ are small. The histogram of the differences $KL\left(g,\hat{g}\right)-KL\left(g,\overset{ˇ}{g}\right)$ is provided in Figure 7. To improve the readability of the figure, a few extreme values on the left are not displayed. From the histogram, we note that, with the exception of the closest regions to 0, the results are symmetric around 0. To compare the two different estimation approaches to the true distribution, we considered the more extreme case on the right, corresponding to a value of 0.0892, for the difference. Then, in Figure 8 (left), we plotted the true CDF, together with the maximum likelihood and AI-based estimated CDFs, along with the points corresponding to the empirical CDF. We note that in this case, the simulated data are far from the true distribution. Hence, the estimated CDF based on the AI, which is closer to the true CDF compared with the one based on MLE, seems to be more robust to outliers. In Figure 8 (right), we repeated the same analysis with a random selected sample (which is more representative of the underlying true model) and observe that the CDFs estimated using the MLE and AI are indistinguishable.

In case of real data, where the underlying PDF *g* is unknown, the goodness of fit for $\hat{g}$ (maximum likelihood estimated PDF) can be evaluated using $KL(\tilde{g},\hat{g})$, i.e., by comparing $\hat{g}$ to the estimated *g* based on the kernel density approach of Section 4. However, this is not a fair tool for comparing $\hat{g}$ to $\overset{ˇ}{g}$, since the AI-based estimated PDF $\overset{ˇ}{g}$ is based on the kernel-based estimation $\tilde{g}$. For this reason, we searched for a different comparison tool.

In order to make a non-parametric comparison of the distributions with parameters given by the AI and the MLE, we considered a different type of KL divergence proposed by Park et al. [32] with properties that have been further studied by Di Crescenzo and Longobardi [33]. This measure is known as the cumulative Kullback–Leibler divergence, and for two non-negative random variables *X* and *Y* with cumulative distribution functions $F_{X}$ and $F_{Y}$, respectively, it is defined by

$$C_{KL}\left(F_{X},F_{Y}\right)=\int_{0}^{+\infty }F_{X}\left(x\right)\log\frac{F_{X}\left(x\right)}{F_{Y}\left(x\right)}dx+E\left(X\right)-E\left(Y\right).$$

Hence, we can use this measure to make comparisons using $F_{e}$, the empirical CDF of the data, in place of $F_{X}$, i.e.,

$$F_{e}\left(t\right)=\frac{1}{n}\sum_{i=1}^{n}I_{\left(T_{i}\leq t\right)},$$

with the corresponding mean given by

$$E\left(X\right)=\frac{1}{n}\sum_{i=1}^{n}T_{i}.$$

In place of $F_{Y}$ we set the MLE-based or AI-based estimate of the CDF *G* of the exponential SSALT model. That is, $\hat{G}\left(t\right)=G\left(t|{\hat{\theta }}_{1},{\hat{\theta }}_{2}\right)$, t>0, with *G* defined in (2), or $\overset{ˇ}{G}$, which is defined analogously. The mean of a CE SSALT exponential model is evaluated as follows:

$$E\left(T\right)=\int_{0}^{\tau }\frac{t}{\theta_{1}}\exp\left(-\frac{t}{\theta_{1}}\right)dt+\int_{\tau }^{+\infty }\frac{t}{\theta_{2}}\exp\left(-\frac{1}{\theta_{2}}\left(t+\frac{\theta_{2}}{\theta_{1}}\tau -\tau \right)\right)dt,$$

which, after integration by parts, is equal to

$$E\left(T\right)=\theta_{1}\left(1-\exp\left(-\frac{\tau }{\theta_{1}}\right)\right)+\theta_{2}\exp\left(-\frac{\tau }{\theta_{1}}\right).$$

Then, denoting by $T_{0}=0$ and $T_{n+1:n}=+\infty$, the cumulative Kullback–Leibler divergence between the considered distribution is evaluated as follows:

$$\begin{array}{ccc} C_{KL}\left(F_{e},\hat{G}\right) & = & \sum_{i=1}^{n_{1}}\int_{T_{i-1:n}}^{T_{i:n}}F_{e}\left(x\right)\log\frac{F_{e}\left(x\right)}{\hat{G}\left(x\right)}dx+\int_{T_{n_{1}:n}}^{\tau }F_{e}\left(x\right)\log\frac{F_{e}\left(x\right)}{\hat{G}\left(x\right)}dx\\ & & +\int_{\tau }^{T_{n_{1}+1:n}}F_{e}\left(x\right)\log\frac{F_{e}\left(x\right)}{\hat{G}\left(x\right)}dx+\sum_{i=n_{1}+2}^{n+1}\int_{T_{i-1:n}}^{T_{i:n}}F_{e}\left(x\right)\log\frac{F_{e}\left(x\right)}{\hat{G}\left(x\right)}dx\\ & & +E(X)-E(T)\\ & = & \sum_{i=2}^{n_{1}}\frac{i-1}{n}\int_{T_{i-1:n}}^{T_{i:n}}\log\left(\frac{i-1}{n}\right)-\log{\hat{G}}_{1}\left(x\right)dx\\ & & +\frac{n_{1}}{n}\int_{T_{n_{1}:n}}^{\tau }\log\left(\frac{n_{1}}{n}\right)-\log{\hat{G}}_{1}\left(x\right)dx\\ & & +\frac{n_{1}}{n}\int_{\tau }^{T_{n_{1}+1:n}}\log\left(\frac{n_{1}}{n}\right)-\log{\hat{G}}_{2}\left(x\right)dx\\ & & +\sum_{i=n_{1}+2}^{n}\frac{i-1}{n}\int_{T_{i-1:n}}^{T_{i:n}}\log\left(\frac{i-1}{n}\right)-\log{\hat{G}}_{2}\left(x\right)dx\\ & & -\int_{T_{n:n}}^{+\infty }\log{\hat{G}}_{2}\left(x\right)dx+E\left(X\right)-E\left(T\right)\\ & = & \sum_{i=2}^{n}\frac{i-1}{n}\log\left(\frac{i-1}{n}\right)\left(T_{i:n}-T_{i-1:n}\right)-\sum_{i=2}^{n_{1}}\frac{i-1}{n}\int_{T_{i-1:n}}^{T_{i:n}}\log{\hat{G}}_{1}\left(x\right)dx\\ & & -\frac{n_{1}}{n}\int_{T_{n_{1}:n}}^{\tau }\log{\hat{G}}_{1}\left(x\right)dx-\frac{n_{1}}{n}\int_{\tau }^{T_{n_{1}+1:n}}\log{\hat{G}}_{2}\left(x\right)dx\\ & & -\sum_{i=n_{1}+2}^{n}\frac{i-1}{n}\int_{T_{i-1:n}}^{T_{i:n}}\log{\hat{G}}_{2}\left(x\right)dx-\int_{T_{n:n}}^{+\infty }\log{\hat{G}}_{2}\left(x\right)dx+E\left(X\right)-E\left(T\right). \end{array}$$

Considering again the simulation study described in Example 2, we verify that the inequality

$$C_{KL}\left(F_{e},\overset{ˇ}{G}\right)<C_{KL}\left(F_{e},\hat{G}\right)$$

is satisfied in 31.51% of the cases.

## 6. Discussion

In this work, we extended the concept of aging intensity to the setup of SSALT models. This provides new insights to the model and enables a deeper understanding of the aging process of the testing units exposed under a step-stress ALT experiment, as well as further clarification of the differences between the CE and TFR models. Based on the AI function, new estimators for the parameters of a SSALT model were proposed and compared to the MLEs in terms of simulated examples and a simulation study. The models and methods discussed mainly referred to simple SSALT experiments with m=2 stress levels, but they are extendable to cases with m>2, as shown in Example 3 for the case m=3.

This approach opens new research directions in SSALT modeling. Additional studies are required to investigate, in depth and with various setups, the performance of the AI-based estimation in comparison to that of the maximum likelihood estimation. Furthermore, alternative estimators could be considered by replacing the distance $H^{*}$ used in Section 4 with another divergence measure, e.g., the KL divergence. Furthermore, one could proceed to interval the estimation of the parameters (asymptotic or bootstrap) and to test the hypothesis on the SSALT model’s parameters (e.g., $\theta_{s+1}=c\theta_{s}$, s=1,…,m−1, for a fixed c∈(0,1)). It is of interest to investigate the robustness of AI-based estimation in comparison to that of the MLE. Finally, it is worth it to explore possible AI-based criteria for optimal SSALT experimental designs.

In summary, further extensive studies are required to investigate the AI-based estimation with various setups.

## Figures and Tables

**Table 1:** Mean and standard deviation of the fitted (MLE) normal distributions for the estimators of $\theta_{1}=33$ and $\theta_{2}=7$ of the SSALT model (2) based on AI and MLE in the simulation study of Example 2 with 10.000 replications.

	Mean	Standard Deviation
${\overset{ˇ}{\theta }}_{1}$	25.6008	4.9854
${\hat{\theta }}_{1}$	24.8684	4.3003
${\overset{ˇ}{\theta }}_{2}$	6.9302	1.3083
${\hat{\theta }}_{2}$	6.9849	1.1683

**Table 2:** Mean and standard deviation of the fitted (MLE) normal distributions for the estimators of $\theta_{1}=33$, $\theta_{2}=7$, and $\theta_{3}=3$ based on AI and MLE in the simulation study of Example 3 with 10.000 replications.

	Mean	Standard Deviation
${\overset{ˇ}{\theta }}_{1}$	29.2562	9.2000
${\hat{\theta }}_{1}$	24.9046	4.3508
${\overset{ˇ}{\theta }}_{2}$	6.4401	2.0313
${\hat{\theta }}_{2}$	7.2282	1.6811
${\overset{ˇ}{\theta }}_{3}$	2.7728	0.9291
${\hat{\theta }}_{3}$	2.9939	0.7604

## Data Availability

This article does not contain data; the examples are simulated and details of the simulation setup are provided.

## Abbreviations

The following abbreviations are used in this manuscript:

AI	Aging Intensity;
ALT	Accelerated Life Testing;
CDF	Cumulative Density Function;
HR	Hazard Rate;
PDF	Probability Density Function;
SSALT	Step-Stress ALT;
SV	Survival Function;
KL	Kullback–Leibler.

## References

[1] Nelson W. (1990). Accelerated Testing: Statistical Models, Test Plans, and Data Analysis.

[2] Meeker W.Q., Escobar L.A. (1998). Statistical Methods for Reliability Data.

[3] Bagdonavicius V., Nikulin M. (2002). Accelerated Life Models: Modeling and Statistical Analysis.

[4] Nelson W. (2005). A bibliography of accelerated test plans. IEEE Trans. Reliab..

[5] Yang G.B. (1994). Optimum constant-stress accelerated life-test plans. IEEE Trans. Reliab..

[6] Miller R., Nelson W. (1983). Optimum simple step-stress plans for accelerated life testing. IEEE Trans. Reliab..

[7] Han D., Ng H.K.T. (2013). Comparison between constant-stress and step-stress accelerated life tests under time constraint. Nav. Res. Logist. (NRL).

[8] Chen L.S., Liang T., Yang M.C. (2022). Designing Bayesian sampling plans for simple step-stress of accelerated life test on censored data. J. Stat. Comput. Simul..

[9] Kohl C., Kateri M. (2019). Bayesian analysis for step-stress accelerated life testing under progressive interval censoring. Appl. Stoch. Model. Bus. Ind..

[10] Lee J., Pan R. (2012). Bayesian analysis of step-stress accelerated life test with exponential distribution. Qual. Reliab. Eng. Int..

[11] Van Dorp J.R., Mazzuchi T.A., Fornell G.E., Pollock L.R. (1996). A Bayes approach to step-stress accelerated life testing. IEEE Trans. Reliab..

[12] Van Dorp J.R., Mazzuchi T.A. (2005). A general Bayes Weibull inference model for accelerated life testing. Reliab. Eng. Syst. Saf..

[13] Sha N., Pan R. (2013). Bayesian analysis for step-stress accelerated life testing using Weibull proportional hazard model. Stat. Pap..

[14] DeGroot M.H., Goel P.K. (1979). Bayesian estimation and optimal designs in partially accelerated life testing. Nav. Res. Logist. Q..

[15] Jiang R., Ji P., Xiao X. (2003). Aging property of unimodal failure rate models. Reliab. Eng. Syst. Saf..

[16] Nelson W. (1980). Accelerated life testing: Step-stress models and data analysis. IEEE Trans. Reliab..

[17] Gouno E., Balakrishnan N., Balakrishnan N., Rao C.R. (2001). Step-stress accelerated life test. Handbook of Statistics.

[18] Balakrishnan N. (2009). A synthesis of exact inferential results for exponential step-stress models and associated optimal accelerated life-tests. Metrika.

[19] Kateri M., Kamps U. (2017). Hazard rate modeling of step-stress experiments. Annu. Rev. Stat. Its Appl..

[20] Bhattacharyya G.K., Soejoeti Z. (1989). A tampered failure rate model for step-stress accelerated life test. Commun. Stat.-Theory Methods.

[21] Kateri M., Kamps U. (2015). Inference in step-stress models based on failure rates. Stat. Pap..

[22] Szymkowiak M. (2018). Characterizations of Distributions through Aging Intensity. IEEE Trans. Reliab..

[23] Nanda A.K., Bhattacharjee S., Alam S.S. (2007). Properties of aging intensity function. Stat. Probab. Lett..

[24] Bhattacharjee S., Nanda A.K., Misra S.K. (2013). Reliability analysis using ageing intensity function. Stat. Probab. Lett..

[25] Buono F. (2022). Multivariate conditional aging intensity functions and load-sharing models. Hacet. J. Math. Stat..

[26] Buono F., Longobardi M., Szymkowiak M. (2022). On Generalized Reversed Aging Intensity Functions. Ric. Mat..

[27] Giri R.L., Nanda A.K., Dasgupta M., Misra S.K., Bhattacharjee S. (2023). On ageing intensity function of some Weibull models. Commun. Stat.-Theory Methods.

[28] Rezaei M., Khalef V.A. (2014). On the Reversed Average Intensity Order. J. Stat. Res. Iran.

[29] Szymkowiak M. (2018). Generalized Aging Intensity Functions. Reliab. Eng. Syst. Saf..

[30] Silverman B. (1986). Density Estimation for Statistics and Data Analysis.

[31] Kullback S., Leibler R.A. (1951). On information and sufficiency. Ann. Math. Stat..

[32] Park S., Rao M., Shin D.W. (2012). On cumulative residual Kullback-Leibler information. Stat. Probab. Lett..

[33] Di Crescenzo A., Longobardi M. (2015). Some properties and applications of cumulative Kullback–Leibler information. Appl. Stoch. Model. Bus. Ind..

